# Plant-Derived Nanovesicle Enhanced Microribonucleic Acid (MicroRNA) Transfer from Nasal Cavity to the Brain

**DOI:** 10.1021/acs.molpharmaceut.5c00931

**Published:** 2025-10-16

**Authors:** Masakazu Umezawa, Fumiya Suyama, Ken Tachibana, Atsuto Onoda, Ken Takeda

**Affiliations:** 1 Research Institute for Science and Technology, Organization for Research Advancement, Tokyo University of Science, 2641 Yamazaki, Noda, Chiba 278-8510, Japan; 2 Department of Hygienic Chemistry, Graduate School of Pharmaceutical Sciences, Tokyo University of Science, 2641 Yamazaki, Noda, Chiba 278-8510, Japan

**Keywords:** vesicular plant-derived nanoparticles, nasal-to-brain route, microRNA

## Abstract

The nasal-to-brain route for drug delivery is potentially useful for the treatment of brain disorders. Not only chemically modified molecules but also nanosized carrier vesicles, such as cell culture-derived exosomes, as small molecules, could be delivered via this route. The focus of the present study was to evaluate the potential of vesicular plant-derived nanoparticles (PDNPs), which have been identified as multivesicular bodies, for transporting molecules via the nasal-to-brain route. PDNPs were isolated from the edible parts of onion (*Allium cepa*), cherry tomato (*Lycopersicon esculentum* *var. cerasiforme*), Delaware grape (*Vitis labrusca* “*Delaware*”), and grapefruit (*Citrus × paradisi*) using commercial kits without ultracentrifugation. Recombinant exogenous microRNA (cel-miR-39) was encapsulated into PDNPs by electroporation. Cel-miR-39-incorporated PDNPs were administered to adult male C57BL/6J mice via intranasal instillation or intravenous injection, and then tissue samples were collected. Cel-miR-39 transportation efficiency was evaluated by quantitative RT-PCR. Intranasal instillation was found to be more effective than intravenous injection for microRNA delivery to the brain. The onion-derived nanoparticle was the most effective transporter of microRNA to the olfactory bulb and caudal brain. The transportation potential and kinetics of other molecules (therapeutic drugs) using onion-derived nanoparticles are of future interest.

## Graphical abstract

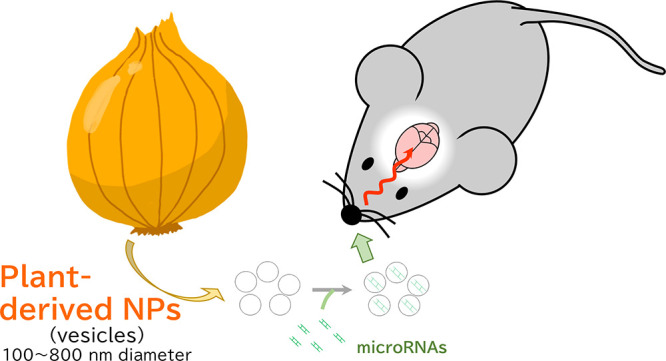

## Introduction

Exosomes are small vesicular nanoparticles, 30–200 nm in diameter, produced by a variety of mammalian cells, and can contain various biomolecules such as proteins, lipids,^1^ small RNAs and microRNAs (miRNAs),^2–4^ mRNA,^5^ and other chemical compounds.^6^ They are generated by endocytosis, endosome sorting into perinuclear multivesicular bodies (MVBs), and subsequent exocytosis of MVBs.^7^ They can also transport molecules from donor cells to recipient cells, thus facilitating cell-to-cell communication^5,8–10^ and regulating the function of recipient cells, which are distant from donor cells.^11,12^

Since nanosized particles released from many different types of mammalian cells have been extensively studied, the establishment of diagnostic and therapeutic strategies using exosomes is expected. Previous studies have reported that exosomes containing a viral membrane glycoprotein can pass through the blood-brain barrier and can deliver drugs and small RNAs to neurons, microglial cells, and oligodendrocytes in the brain.^13^ The nasal delivery of drugs to the brain appears to involve extracellular pathways^14^ and may be useful, although low efficiency and capacity attributable to the limited volume of the nasal cavity could limit the use of this route.^15,16^ The olfactory and the trigeminal pathways connect directly the nasal mucosa to the brain^17^ and thus bypass the blood-brain barrier. In addition, the use of these pathways bypasses the systemic circulation of administered substances and thereby avoids the potential of the following side effects. Thus, it has been hypothesized that nanoparticles may reach the brain through the nasal mucosa.^18–20^ In this context, artificial nanoparticles have been studied as a platform for nose-to-brain drug delivery.^21^ Well-designed nanoparticle formulation is effective for drug delivery via the route to the brain.^22^ Also, intranasally instilled exosomes isolated from the conditioned media of a mouse lymphoma cell line (EL4)^23^ and a mouse macrophage cell line (RAW 264.7)^24^ are able to enter the brain. Encapsulation of drugs by the exosome can protect the brain from lipopolysaccharide (LPS)-induced inflammation^23^ and the symptoms of Parkinson’s disease.^24^ Thus, exosomes are potentially good delivery vectors for drugs, proteins, and nucleic acids to brain cells. The efficacy of nanoparticulate drug delivery systems in the nasal cavity and central nervous system has not been extensively studied,^25^ and still needs to be carefully considered.

Exosome-like vesicles were recently found in the paramural spaces of plants.^26^ These secretory vesicles play a role during viral and fungal infections.^26,27^ Subsequent studies succeeded in isolating nanosized vesicles from grapes,^28,29^ grapefruit,^29–31^ carrot,^29^ and ginger^29^ using ultracentrifugation followed by purification on a sucrose gradient. These nanoparticles were confirmed to be taken up by intestinal macrophages,^29,31^ stem cells,^29^ cultured epithelial cells,^28^ and tumor model lesions.^30^ Since the vector capacity of exosomes and similar vesicles is dependent on surface modifications and administration route, it is important to compare small molecule transfer by exosomes and vesicles derived from different organisms. The present study focused on plant-derived vesicles because they present no possibility of serum contamination, although the quality of commercially available products is potentially not standardized. In the present study, nanosized vesicles were obtained from plants using an ultracentrifugation-free procedure in order to preserve nanoparticle shape and function, and their capabilities for brain tissue penetration were compared.

## Materials and Methods

### Isolation of Nanovesicles from Plants

Grapefruit (*Citrus × paradisi*), onion (*Allium cepa*), Delaware grapes (*Vitis labrusca* “*Delaware*”), and cherry tomatoes (*Lycopersicon esculentum* *var. cerasiforme*) were purchased from a food market, washed with water, and stored at – 20 °C. The skin was removed from grapefruits and onions before freezing. After the mixture returned to 20 °C, the Delaware grape skin was removed, and each plant was grated to obtain juice. The juice was sequentially centrifuged at 2000*g* for 20 min and 10,000*g* for 60 min at 4 °C to remove large debris. The treated juice from onion, grape, and cherry tomatoes was filtered through a 450 nm filter (Advantec Co., Ltd., Tokyo, Japan). The supernatants were then treated with the total exosome isolation kit (Thermo Fisher Scientific Inc.) according to the manufacturer’s instructions in order to obtain pellet exosome-like vesicles. The resulting pellets containing plant-derived nanoparticles (PDNPs) from each species were resuspended in 25 μL of phosphate-buffered saline (PBS) and purified by exosome spin columns (MW3000, Thermo Fisher Scientific Inc.).

### Isolation of Exosomes from Cell Culture Media

A mouse macrophage cell line (RAW 264.7) was maintained in RPMI 1640 media (Wako Pure Chemical Industries, Ltd.) supplemented with 10% (v/v) heat-inactivated fetal bovine serum (FBS) and 100 U/mL penicillin with 100 μg/mL streptomycin (Sigma-Aldrich) in a humidified CO_2_ incubator at 37 °C. Culture medium was replaced with RPMI 1640 media with antibiotics and 10% (v/v) exosome-depleted FBS (Funakoshi) 24 h prior to exosome isolation. Culture supernatants were collected and treated with the Total Exosome Isolation kit (Thermo Fisher Scientific Inc.) according to the manufacturer’s instructions to pellet exosome fractions. The resulting pellets were resuspended in PBS (25 μL).

### Transmission Electron Microscopy

For electron microscopic analysis, the samples were fixed with 2% formaldehyde for 30 min on ice, placed on a collodion-coated 200 Cu mesh (Nisshin EM), air-dried, washed with water for 5 min, stained with uranyl acetate for 7 min, washed with water, dried overnight, and then observed under transmission electron microscopy (TEM; JEM1200EXII, JEOL Ltd.) at an 80 kV accelerating voltage.

### Dynamic Light Scattering

The size distribution of the plant-derived vesicles and RAW 264.7-derived exosomes was determined by dynamic light scattering (DLS) using a NANO-ZS (Sysmex Co., Kobe, Hyogo, Japan). For DLS measurement, RAW264.7 cell-derived exosomes and PDNPs were diluted to 0.1 and 0.5 μg of protein/μL in PBS, respectively.

### Protein Analysis

Vesicle and exosome suspensions were dissolved in RIPA buffer [10 mM Tris-HCl (pH 7.4), 150 mM NaCl, 1 mM EDTA (pH 8.0), 0.1% SDS, 1% Nonidet P-40, 0.1% sodium deoxycholate, and protease inhibitor cocktail (Complete, EDTA-free, Roche Diagnostics)], incubated for 10 min on ice, and centrifuged at 8000*g* for 10 min at 4 °C. Supernatant protein were serially diluted up to 1000 times, and the protein concentrations were analyzed using the BCA protein assay kit (Thermo Fisher Scientific). Protein samples (1 μg) were separated by SDS-PAGE and detected by using EzStain Silver (Atto Co., Tokyo, Japan) according to the manufacturer’s instructions.

### Fluorescence Microscopy

Plant-derived vesicles and RAW 264.7-derived exosomes were incubated with 20 μM PKH26 fluorescent dye (Sigma-Aldrich) for 5 min at room temperature. Excess PKH26 dye was removed using exosome spin columns (MW3000; Thermo Fisher Scientific). PKH26-labeled samples were added to RAW 264.7 cell culture medium and observed 1 h later using a BZ-9000 fluorescence microscope (Keyence Co., Osaka, Japan).

### Introducing Exogenous MicroRNA to Nanovesicles by Electroporation

5′-Biotinylated *C. elegans* miRNA-39 (biotin-cel-miR-39) was synthesized by Hokkaido System Science Co., Inc. (Sapporo, Hokkaido, Japan) with the following sequences: 5′-biotin-UCACCGGGUGUAAAUCAGCUUG (cel-miR-39–5p) and 5′-biotin-AGCUGAUUUCGUCUUGGUAAUA (cel-miR-39–3p). Biotin-cel-miR-39 (1.5 nmol each) was heated with annealing buffer [10 mM Tris-HCl (pH 8.0), 20 mM NaCl] for 60 s at 90 °C and then gradually cooled for 60 min to 20 °C. Annealed biotin-cel-miR-39 (0.4 nmol each) was mixed with plant-derived vesicles (containing 2 mg of protein) or RAW 264.7 exosomes (0.4 mg of protein), diluted to 400 μL with PBS, and then electroporated (400 V, 500 μF) in a 0.4 cm gap electroporation cuvette (Bio-Rad Laboratories, Inc., TX, USA) using a Gene Pulser Xcell (Bio-Rad Laboratories, Inc.). Mixtures of the vesicles or exosomes with biotin-cel-miR-39 were also prepared as a nonelectroporation control.

### Administration of Nanovesicles with Exogenous MicroRNA to Mice

Adult male C57BL/6J mice (10–19 weeks of age; SLC Inc., Shizuoka, Japan, or CLEA Japan, Inc., Tokyo, Japan) were housed under controlled temperatures (23 ± 1 °C) and humidity (55% ± 5%) with a 12 h dark/light cycle and *ad libitum* access to food and water. All animals were treated and handled in accordance with the national guidelines for the care and use of laboratory animals and with the approval of the institutional animal care and use committee (approval number: Y15058).

Twenty microliters of plant-derived vesicles (5 mg protein/mL) or RAW 264.7 exosomes (1 mg protein/mL), either mixed or treated with biotin-cel-miR-39, was administered to mice by intranasal instillation. In the intravenous injection study, 100 μL of vesicles or exosomes (same concentration as the case of intranasal instillation to increase the dose for the injection) was administered to mice via the tail vein. Sixty minutes later, the brain was collected and divided into olfactory bulb, rostral, and caudal regions (cut at the lambda) under an overdose of pentobarbital via intraperitoneal injection. The lungs and livers were also collected. All tissue samples were immediately frozen in liquid nitrogen and stored at −80 °C until use.

### Ribonucleic Acid Isolation and Real-Time PCR

The tissue samples were homogenized in lysis buffer [1 mL for dissected brain (<200 mg), 200 μL for a small pieces (<40 mg) of liver and lung] composed of 20 mM Tris-HCl (pH 7.5), 200 mM NaCl, 2.5 mM MgCl_2_, 0.05% Nonidet P-40, 1 U/L SUPERase In RNase Inhibitor (Life Technologies), 1 mM dithiothreitol, and protease inhibitor cocktail (Complete, EDTA-free, Roche Diagnostics) by Bio Masher II (Nippi, Inc., Tokyo, Japan). After homogenization, samples were centrifuged at 21,500*g* for 5 min at 4 °C. Dynabeads M-270 streptavidin (150 μg, Veritas Co., Tokyo, Japan) was added to the supernatant of tissue homogenates in order to capture biotin-cel-miR-39. The beads were then collected with a magnetic rack, washed three times with lysis buffer, and then suspended in 5 μL of distilled water. After heating at 95 °C for 5 min, the suspensions were treated at 42 °C for 60 min with Universal cDNA Synthesis Kit (Exiqon, Denmark) according to the manufacturer’s instructions, followed by further heating at 95 °C for 5 min and immediate cooling on ice. A magnetic rack was then used to remove streptavidin beads and obtain complementary DNA solutions. The solutions were mixed with SYBR Green Master Mix (Thunderbird; Toyobo, Osaka, Japan) and specific primers for cel-miR-39–3p (miRCURY LNA PCR Primer Set; Exiqon), and then analyzed using an Mx3000P Real-Time PCR System (Agilent Technologies) with an initial hold step (95 °C for 60 s) and 50 cycles of a two-step PCR procedure (95 °C for 15 s and 60 °C for 60 s).

### Statistical Analysis

The difference in cel-miR-39 detection levels in each group was analyzed by one-way analysis of variance followed by the Tukey–Kramer test. Significance was determined as *P* < 0.05.

## Results

### Characterization of Nanovesicles from Plants and RAW264.7 Exosomes

Electron microscopy confirmed the isolation of nanosized vesicles from RAW 264.7 cell culture medium and plant body extracts. Exosomes from the RAW 264.7 cell culture medium were generally isolated as nanovesicles with a diameter ranging from 30 to 200 nm (Figure 1E). Nanovesicles of similar or larger sizes were isolated from onion (diameter distribution peaks as determined by 105.7 and 712.4 nm; Figure 1A), cherry tomato (141.8 nm; Figure 1B), Delaware grape (531.2 nm; Figure 1C), and grapefruit (164.2 nm; Figure 1D) extracts. SDS-PAGE showed that the proteins contained within PDNPs from different donor plants were different (Figure 1F).

**Figure 1:**
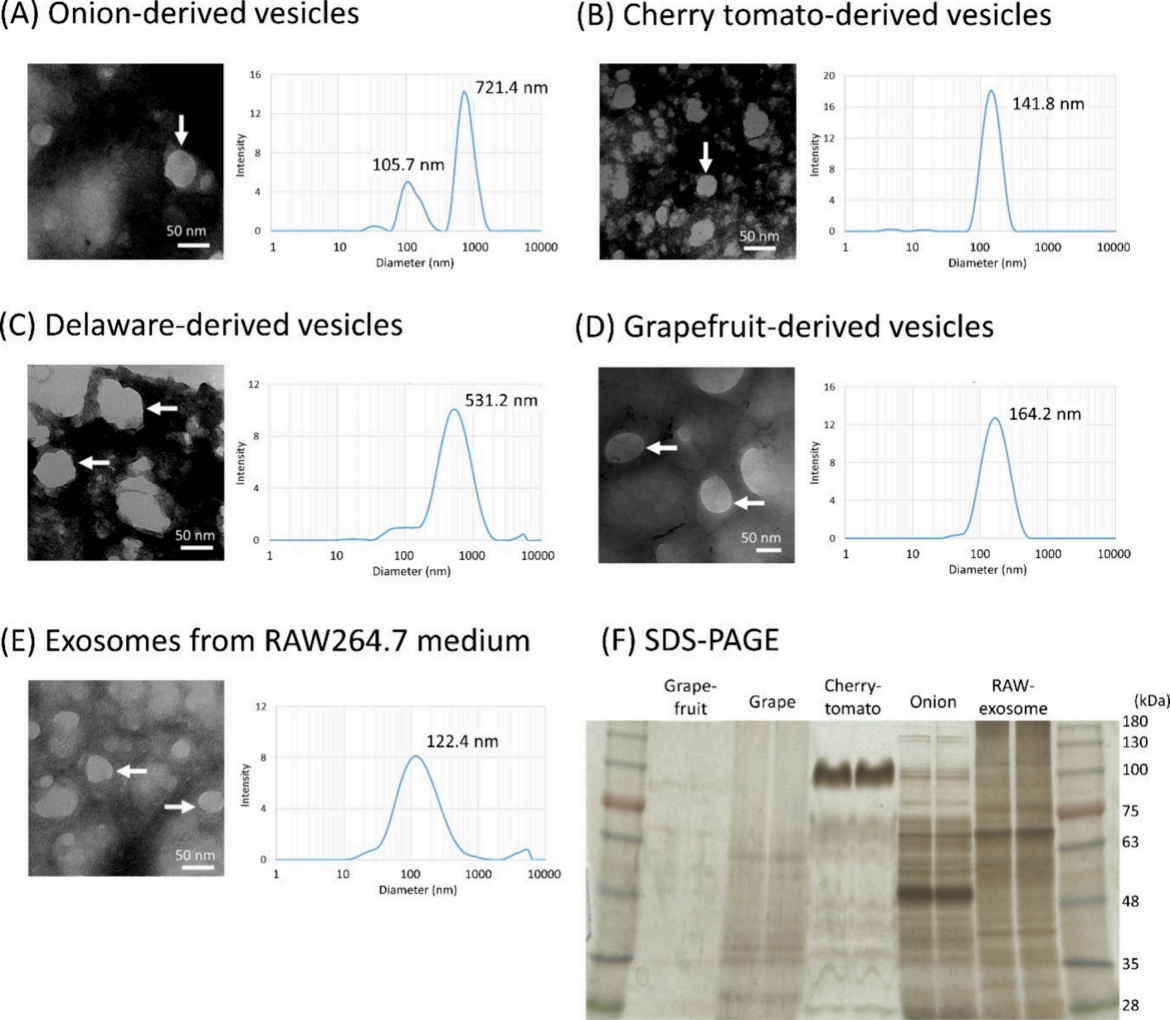
Characterization of nanovesicles isolated from plant extracts and RAW 264.7 cell culture medium exosomes. Transmission electron microscopic (TEM) images and dynamic light scattering (DLS) data of nanovesicles from (A) onion, (B) cherry tomato, (C) Delaware grapes, and (D) grapefruit extracts, and (E) exosomes from RAW 264.7 cell culture medium are shown. (F) Segregation image of nanovesicular and exosomal protein obtained by sodium dodecyl sulfate-polyacrylamide gel electrophoresis (SDS-PAGE).

### Uptake of Plant-Derived Nanovesicles and RAW 264.7 Exosomes by Cultured Cells

It is well-known that exosomes are taken up by various kinds of cells. Similarly, PDNPs were taken up by RAW 264.7 cells during the 60 min incubation in culture medium, as seen in fluorescent microscopic images of PKH26-labeled vesicles (Figure 2). PKH26 signals on the vesicle membrane did not diffuse upon RAW 264.7 cell membranes but were detected as small dots in the cytoplasm. These images suggest that PDNPs were taken up through endocytosis rather than through cell membrane fusion. Cellular images of the negative (vesicle-free) control showed no fluorescence, suggesting that excess PKH26 dye was removed by the spin-column method.

**Figure 2:**
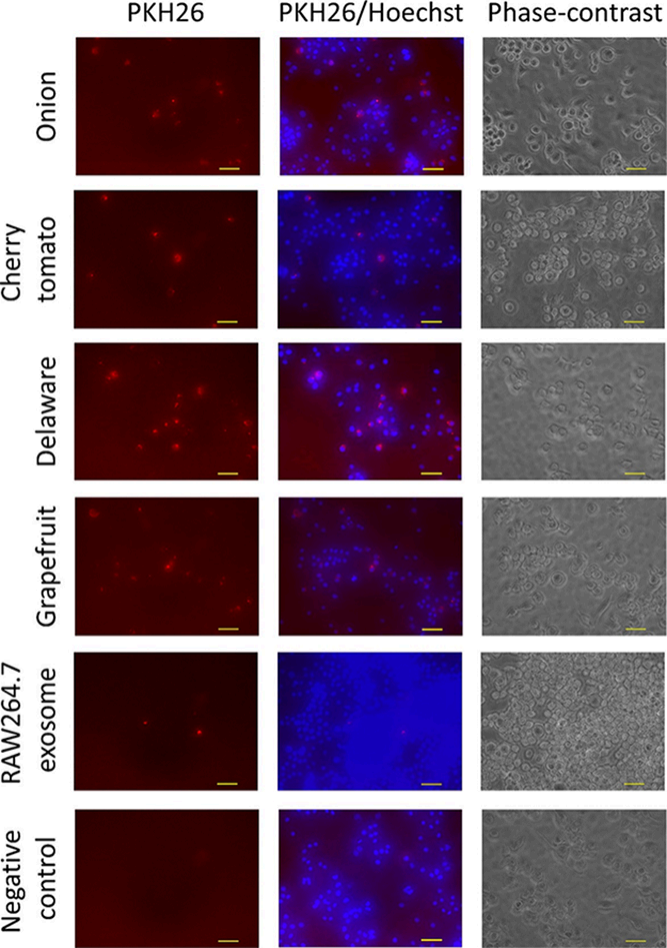
Cellular uptake of nanovesicles isolated from plant extracts and RAW 264.7 cell culture medium exosomes. Plant-derived nanovesicles and RAW 264.7 exosomes were labeled using PKH26, a lipid membrane labeling dye. After excess dye was removed by exosome spin columns (MW 3000), labeled vesicles and exosomes were added to the RAW 264.7 cell culture medium and incubated for 60 min. Fluorescent images (excitation and emission wavelengths: 545 and 605 nm, respectively) of cells treated with PDNPs derived from onion, cherry tomato, Delaware grape, and grapefruit, exosomes from RAW 264.7 culture medium, and the control (no vesicles or exosomes) were captured by microscopy.

### Transport of Exogenous MicroRNA from Nasal Cavity to the Brain by Exosomes and Plant-Derived Nanovesicles

Biotin-cel-miR-39 mixed with RAW 264.7 exosomes with or without electroporation was administered to mice intranasally or intravenously. The microRNA distribution was evaluated by using quantitative RT-PCR analysis of dissected brain, liver, and lung tissue homogenates. Biocel-miR-39 was primarily distributed in the brain, especially in the olfactory bulb, following intranasal instillation (Figure 3A), while it was found in both the brain and liver after intravenous injection (Figure 3B). The detection amount was 38-, 118-, and 88-fold higher in the olfactory bulb, rostral brain, and caudal brain, respectively, relative to that in the liver after intranasal instillation, whereas it was 3.4-, 0.3-, and 2.0-fold higher in the olfactory bulb, rostral brain, and caudal brain, respectively, compared to that in the liver after intravenous injection. Intranasal instillation was, therefore, the more effective administration route for microRNA delivery to the brain. Unexpectedly, the miRNA transport to a part of the brain, including the olfactory bulb, was higher per organ weight compared to that to the liver. However, because the liver has a larger weight, the results did not mean that the total miRNA transport to the brain was large following intravenous administration. Very little biotin-cel-miR-39 was detected in murine organs after administration of nonelectroporated exosomes, but intranasal instillation still yielded higher results than intravenous injection did. This suggests that the microRNA is degraded in the blood, possibly by RNases, if not encapsulated by exosomes, but remains relatively stable throughout nasal-to-brain transmission. Our results showed that exosomes were effective for the nasal-to-brain delivery of miRNA even without electroporation (Figure 3A), suggesting that miRNA adsorption onto exosomes alone may also work for the delivery. However, since the vertical axes of Figure 3A,B differ 10 times, the RAW 264.7 cell-derived exosomes were not effective for the nasal-to-brain miRNA transport. We then investigated the potential for transport using PDNPs.

**Figure 3:**
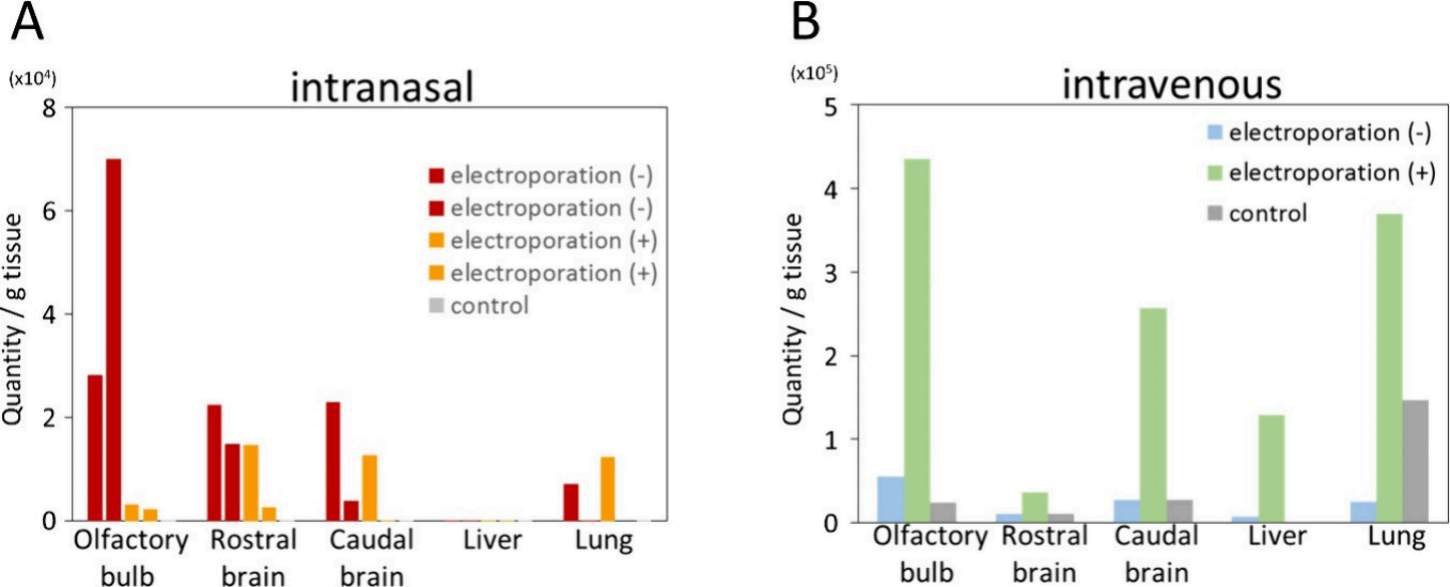
Cel-miR-39 levels in the murine brain, liver, and lung after administration of RAW 264.7 cell culture medium exosomes. Cel-miR-39 levels in tissue samples (*n* = 2/group) were evaluated using quantitative RT-PCR. Detected levels (relative quantity per tissue weight) at 1 h after (A) intranasal instillation and (B) intravenous injection of biotin-cel-miR-39 treated/mixed with exosomes are shown. Exosome-free sample (biotin-cel-miR-39 alone) was administered as a control.

The transfer of biotin-cel-miR-39 to murine brain regions by mixing (without electroporation) or encapsulating (by electroporation) PDNPs was evaluated at 1 h after intranasal instillation (Figure 4). Cel-miR-39 was not detected in any sample after microRNA incorporation with grapefruit-derived nanovesicles. Our protocol using the total exosome isolation kit is not likely to be suitable for extracting PDNPs from grapefruit, which showed the absence of protein bands in the extract (Figure 1F). MicroRNA transfer was enhanced by PDNP encapsulation, suggesting that PDNPs are effective carriers for the delivery of microRNAs from the nasal cavity to the brain. The scales of the vertical axes in Figures 3A and 4 differ 100 times, and the nasal-to-brain miRNA delivery by onion- and cherry tomato-derived PDNPs showed >50 times larger than that by the RAW 264.7-derived exosomes. Interestingly, cel-miR-39 levels were high in the caudal brain after delivery via onion-derived nanoparticles (>30 times larger than the RAW 264.7-derived exosomes). It should also be noted that microRNA levels tended to be high in the olfactory bulb after the administration of PDNPs derived from onion or cherry tomato. MicroRNA detection levels increased in the rostral brain after encapsulation with Delaware grape-derived nanoparticles.

**Figure 4:**
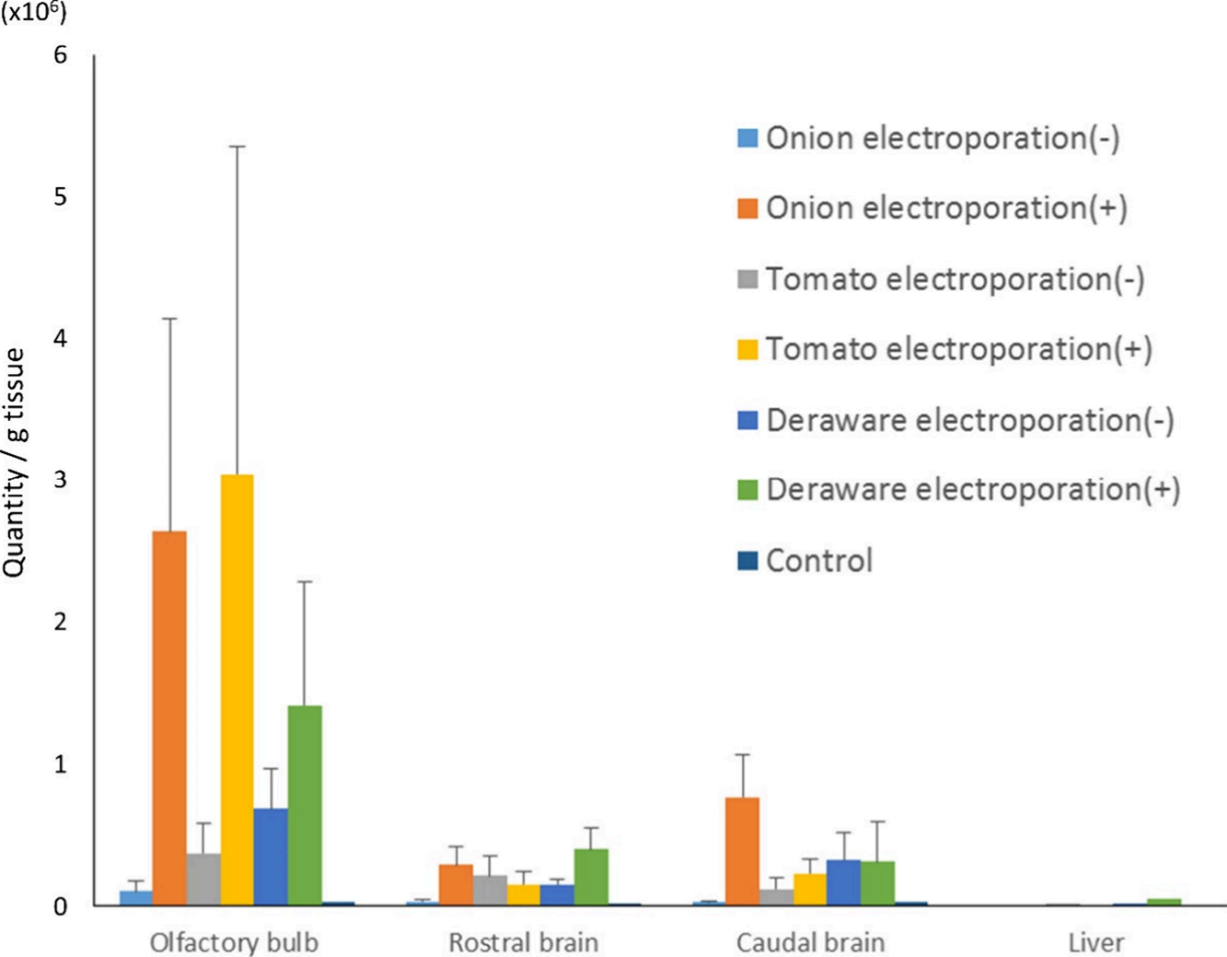
Cel-miR-39 levels in murine dissected brain and liver after intranasal instillation with plant-derived nanoparticles. Cel-miR-39 levels present in each tissue sample were examined by quantitative RT-PCR. Levels (relative quantity per tissue weight) at 1 h after intranasal instillation of biotin-cel-miR-39 treated/mixed with exosomes or plant-derived nanoparticles are shown. Data are shown as mean ± SEM (*n* = 3/group). Vesicle-free sample (biotin-cel-miR-39 alone) was administered as a control.

## Discussion

Drug delivery to the brain has been a major challenge because of difficulties in penetrating the blood-brain barrier.^32–34^ Intranasally administered small molecules (including siRNAs) are effective in reaching the brain.^35^ Encapsulating these molecules using vesicles may further improve their efficiency.^13^ Contrary to the results of previous studies,^28–31^ the present study showed that nanosized PDNPs could be isolated without ultracentrifugation. Although we have no data on the isolation efficiency to be compared, our protocol using the Exosome Isolation kit successfully collected PDNPs containing more than several micrograms of proteins from several tens of grams of plant samples. Further investigations are needed to determine the number concentration and its relation to the protein content of collected PDNPs to clarify the isolation efficiency. Although the PDNPs did not decrease the cell viability, their detailed toxicity using functional biomarkers should be investigated in further studies. The uptake of dye-labeled PDNPs by cultured cells was observed by fluorescent microscopy. The confirmation of cellular uptake requires confocal microscopy, but is not the major target in the present study. Rather, PDNPs were used to evaluate the delivery of microRNA from the nasal cavity to the brain in mice. PDNPs were isolated from juices prepared by grinding various plants and therefore contained not only multivesicular bodies but also reconstituted micelles derived from cell membranes and subcellular organelles. Some molecules related to intracellular transport may be expressed on the vesicular PDNP membrane.

Chemical compounds can cross the olfactory pathway and reach the brain from the nasal cavity via a transcellular pathway involving receptor-mediated endocytosis followed by passive diffusion, a paracellular pathway through open clefts in the cell membrane, and direct transport primarily to the olfactory bulb through olfactory neuron cells by intracellular axonal transport.^36^ Previous studies have shown that inorganic nanoparticles can be transferred to the olfactory bulb within 1 h after intranasal instillation.^37^ However, nanoscale vesicular exosomes are primarily distributed in the liver and spleen after entering the whole body circulation via intravenous injection.^10,38,39^ Similarly, biotin-cel-miR-39 encapsulated within RAW 264.7-derived exosomes was primarily distributed in the liver as well as the lung within 1 h after intravenous injection. The microRNA distribution in the brain after intravenous injection suggested that exosomes could penetrate the blood-brain barrier, as also shown in a previous study.^24^ Because olfactory epithelia in the nasal cavity are close to the olfactory bulb of the brain, they represent a potentially useful and effective administration route for substances and drugs to the brain with decreased nonspecific distribution to other organs. Nanosized exosomes (diameter 30–100 nm) isolated from mouse cell cultures and administered to the murine nasal cavity were found in the brain within 30 min, whereas similar microsized particles (diameter 500–1000 nm) were not able to enter the brain.^23^ Similarly, microRNA transfer to the olfactory bulb using PDNPs from Delaware grapes, which showed larger aerodynamic diameters, tended to be less than when PDNPs from onion or cherry tomatoes were used. PDNPs of 30–150 nm may be effective for microRNA and small molecule transport from the nasal cavity to the brain.

Although low efficiency and capacity attributable to the limited volume of the nasal cavity present issues,^16^ some molecules and PDNPs composed of lipid and proteins are more stable in the nasal-to-brain route than in the circulation, as demonstrated by the brain distribution profile of cel-miR-39 mixed with RAW 264.7 exosomes without electroporation. Our current results showed that miRNA delivery to the brain via intranasal administration was achieved using a 20% dose of that required for intravenous administration. In the present study, 5 times more vesicles (PDNPs; based on the protein amount) were used to encapsulate 0.4 nmol of miRNA than the exosomes because larger amounts of PDNPs were easily obtained from the plant samples. The results showed a higher delivery capacity of PDNPs for miRNA compared to the exosomes, but potentially lower loading density. Previous studies have shown that a designed biodegradable dendrimer^35^ and a specific peptide (TAT/MGF)-tagged PEGylated chitosan nanoparticle^40^ were effective in siRNA nasal-to-brain delivery. PDNPs may present a good drug delivery vector to the central nervous system via intranasal instillation. MicroRNA incorporated with PDNPs showed different distribution patterns in the brain compared to the report for microRNA within RAW 264.7 exosomes after intranasal instillation, possibly owing to a difference in size and chemical composition (proteins, sugar-chains, among others) on their surface, which are expected to be investigated from DLS and SDS-PAGE data in this study. This study also has a limitation of the lack of longer-term distribution and metabolic fate of delivered miRNAs. Points of interest for future work include characterization of time-dependent changes in PDNP-mediated microRNA distribution after intranasal instillation, as well as potentially critical factors for determining and controlling the transport patterns of small RNAs and other molecules. Such points have still been investigated and will be reported with other loaded molecules, such as functional peptides, with their pharmacological effect data in future works. The additional information will contribute to the development of drug delivery methods from the nasal cavity to the central nervous system.

## 3

Department of Medical and Robotic Engineering Design, Faculty of Advanced Engineering, Tokyo University of Science, 6–3–1 Niijuku, Katsushika, Tokyo 125–8585, Japan (M.U.)

## 4

Faculty of Pharmaceutical Sciences, Sanyo-Onoda City University, 1–1–1 University Street, Sanyo-Onoda city, Yamaguchi 756–0884, Japan (K.Tachibana, A.O., and K.Takeda).
